# Identifying a nasal gene expression signature associated with hyperinflation and treatment response in severe COPD

**DOI:** 10.1038/s41598-020-72551-0

**Published:** 2020-10-15

**Authors:** Alen Faiz, Kai Imkamp, Erica van der Wiel, Ilse M. Boudewijn, Gerard H. Koppelman, Corry-Anke Brandsma, Huib A. M. Kerstjens, Wim Timens, Sebastiaan Vroegop, Henk R. Pasma, Wim G. Boersma, Pascal Wielders, Frank van den Elshout, Khaled Mansour, Katrina Steiling, Avrum Spira, Marc E. Lenburg, Irene H. Heijink, Dirkje S. Postma, Maarten van den Berge

**Affiliations:** 1 Department of Pulmonology, University of Groningen, University Medical Center Groningen, Hanzeplein 1, 9700 RB Groningen, The Netherlands; 2 GRIAC (Groningen Research Institute for Asthma and COPD), University of Groningen, University Medical Center Groningen, Groningen, The Netherlands; 3 Department of Pathology & Medical Biology, Section Medical Biology, University of Groningen, University Medical Center Groningen, Groningen, The Netherlands; 4 Department of Pediatric Pulmonology and Pediatric Allergology, Beatrix Children’s Hospital, University of Groningen, University Medical Center Groningen, Groningen, The Netherlands; 5 Department of Pathology and Medical Biology, University of Groningen, University Medical Center Groningen, Groningen, The Netherlands; 6 Department of Pulmonary Diseases, Martini Hospital Groningen, Groningen, The Netherlands; 7 Department of Pulmonary Diseases, Medical Center Leeuwarden, Leeuwarden, The Netherlands; 8 Department of Pulmonary Diseases, Medical Center Alkmaar, Alkmaar, The Netherlands; 9 Department of Pulmonary Diseases, Catharina Hospital Eindhoven, Eindhoven, The Netherlands; 10 Department of Pulmonary Diseases, Rijnstate Hospital, Arnhem, The Netherlands; 11 Department of Pulmonary Diseases, Orbis Concern, Sittard, The Netherlands; 12 Division of Computational Biomedicine, Department of Medicine, Boston University School of Medicine, Boston, MA USA

**Keywords:** Gene expression, Outcomes research

## Abstract

Hyperinflation contributes to dyspnea intensity in COPD. Little is known about the molecular mechanisms underlying hyperinflation and how inhaled corticosteroids (ICS) affect this important aspect of COPD pathophysiology. To investigate the effect of ICS/long-acting β_2_-agonist (LABA) treatment on both lung function measures of hyperinflation, and the nasal epithelial gene-expression profile in severe COPD. 117 patients were screened and 60 COPD patients entered a 1-month run-in period on low-dose ICS/LABA budesonide/formoterol (BUD/F) 200/6 one inhalation b.i.d. Patients were then randomly assigned to 3-month treatment with either a high dose BDP/F 100/6 two inhalations b.i.d. (n = 31) or BUD/F 200/6 two inhalations b.i.d. (n = 29). Lung function measurements and nasal epithelial gene-expression were assessed before and after 3-month treatment and validated in independent datasets. After 3-month ICS/LABA treatment, residual volume (RV)/total lung capacity (TLC)% predicted was reduced compared to baseline (p < 0.05). We identified a nasal gene-expression signature at screening that associated with higher RV/TLC% predicted values. This signature, decreased by ICS/LABA treatment was enriched for genes associated with increased p53 mediated apoptosis was replicated in bronchial biopsies of COPD patients. Finally, this signature was increased in COPD patients compared to controls in nasal, bronchial and small airways brushings. Short-term ICS/LABA treatment improves RV/TLC% predicted in severe COPD. Furthermore, it decreases the expression of genes involved in the signal transduction by the p53 class mediator, which is a replicable COPD gene expression signature in the upper and lower airways.

Trial registration: ClinicalTrials.gov registration number NCT01351792 (registration date May 11, 2011), ClinicalTrials.gov registration number NCT00848406 (registration date February 20, 2009), ClinicalTrials.gov registration number NCT00158847 (registration date September 12, 2005).

## Introduction

Chronic Obstructive Pulmonary Disease (COPD) is a leading cause of death worldwide^1^. The presence and severity of COPD is generally documented by a decreased forced expiratory volume in 1 s (FEV_1_) and FEV_1_/forced vital capacity (FVC) ratio that is not (fully) reversible with bronchodilation^2^. In addition, air trapping is present in a considerable proportion of patients, as reflected by an increased residual volume (RV) in relation to total lung capacity (TLC). This trapped air, i.e. hyperinflation that is associated with heterogeneity in patency of the small airways, contributes importantly to the dyspnea intensity experienced by COPD patients^3^.

COPD is characterized by an abnormal inflammatory response in the airways and lungs to inhaled noxious gases and particles^4,5^. The inflammation in COPD occurs in the central and peripheral airways and lung parenchyma. A number of mechanisms can link inflammation and hyperinflation in COPD^6^. Inflammation of the airways is associated with (1) oedema which can contribute to increased airway resistance in large and small airways; (2) neutrophil infiltration that is related a.o. to increased oxidative stress, and cytokine and elastase release that associate with airway wall fibrosis and mucus production/plugging^7^ and (3) emphysema contributing to the loss of elastic recoil and (small) airway collapse at expiration^8^. All these components contribute to lung hyperinflation. Little is known about the molecular mechanisms driving the cellular actions in airways and lung parenchyma, underlying the complex pathophysiology of hyperinflation. Inflammation likely contributes to hyperinflation since it has been shown that the ICS fluticasone, with and without LABA treatment can reduce hyperinflation in COPD^9,10^. One of the cell types directly affected by ICS are epithelial cells aligning the airway walls as a direct barrier against inhaled toxic substances^11^. Epithelial cells contribute to inflammation by producing pro-inflammatory cytokines and mediators during exposure to inhaled toxic particles. Smoking has been shown to affect the gene-expression profile in upper and lower airways comparably, suggesting that one may use nasal epithelial brushes as a non-invasive marker for the lower airways^12^.

The study was originally designed to compare the effect of beclomethasone/formoterol (BDP/F) to budesonide/formoterol (BUD/F) treatment in patients with severe COPD and hyperinflation. However, the study was terminated prematurely due to slow inclusion and was thus underpowered. Therefore, we grouped the two treatment arms to analyze the effects of treatment on RV% predicted at day 84 (3 months). As a secondary outcome, we investigated FEV_1_%, TLC% and RV/TLC% predicted. Finally, we performed genome-wide gene-expression profiling in the nasal epithelium to identify a gene-expression signature related to hyperinflation in COPD.

## Methods

### Patient population

The “A 12-week, Multicentre, Randomised, Double-blind, Double-dummy, 2-arm Parallel Group Study Comparing the Efficacy and Safety of Foster^®^ 100/6 (Beclomethasone Dipropionate 100 µg Plus Formoterol 6 µg/Actuation), 2 Puffs b.i.d., Versus Symbicort^®^ 200/6 (Budesonide 200 µg Plus Formoterol 6 µg/Actuation), 2 Inhalations b.i.d., on Parameters of Small Airway Function in Patients With Chronic Obstructive Pulmonary Disease” (FAIR) study (registered as NCT01351792 registration date May 11, 2011) included COPD patients when they fulfilled the following inclusion criteria: age ≥ 40 years, a post-bronchodilator FEV1/FVC < 0.7 and FEV_1_ < 50% of the predicted normal value, and a smoking history of at least 10 pack-years, regular use of bronchodilators, a Functional Residual Capacity (FRC) > 120% of predicted, and a Baseline Dyspnea Index (BDI) total score ≤ 10. We excluded patients with a diagnosis of asthma or other clinically or functionally relevant respiratory disorders (other than COPD). The online supplement presents further details on eligibility criteria. Patients were recruited between September 2011 and February 2013 from different centers in the Netherlands: University Medical Center Groningen (UMCG), Medical Center Leeuwarden, Martini Hospital Groningen, Medical Center Alkmaar, Catharina Hospital Eindhoven, Rijnstate Hospital Arnhem and Orbis Concern Sittard.

**Figure 1:**
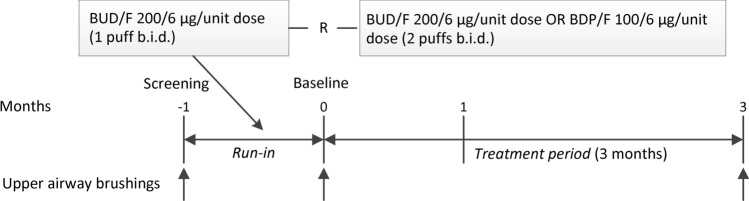
Schematic diagram of FAIR study outline.

A graphical overview of the study is presented in Fig. 1. After inclusion, all patients entered a 1-month run-in period during which their current inhaled treatment was switched to a low dose Symbicort Turbuhaler 200/6 one inhalation b.i.d. (budesonide 200 µg plus formoterol 6 µg/actuation dry powder inhalation (BUD/F), AstraZeneca, London, UK Limited) with as needed salbutamol pressurized metered dose inhaler (pMDI) 100 µg and/or ipratropium bromide 20 µg (pMDI). No other inhaled medications or oral prednisolone were allowed during the study. After the run-in period, patients were randomly assigned to a 3-month treatment period with Foster 100/6 two inhalations b.i.d. (beclomethasone dipropionate 100 µg plus formoterol 6 µg/actuation pressurized metered dose inhaler (BDP/F)), Chiesi Pharmaceutici S.p.A., Parma, Italy) or BUD/F 200/6, two inhalations b.i.d. Lung function measurements and body plethysmography were performed at screening (month − 1), at baseline, and after 1 and 3 months of treatment. Nasal epithelial brushes (CYTO-PAK Cytosoft Brush CP-5B, Medical Packaging Corp., Camarillo, USA), were taken at screening (month − 1), at baseline, and after 3 months of treatment. The methods for mRNA isolation, labelling, microarray hybridization (Affymetrix_HuGene_ST1.0 Array) and quality control were conducted as previously described^13^. The study was approved by the medical ethics committee in Groningen and all subjects gave their written informed consent. All methods were performed in accordance with the relevant guidelines and regulations. The between groups comparison originally planned lacks of statistical power. Nevertheless, collected data are valuable for the purpose of the genome-wide gene-expression profiling and for the investigation of ICS/LABA treatment effect on hyperinflation.

### Statistical analysis

In brief, the lung function changes with treatment were analyzed using a linear mixed effects model with time defined as a categorical variable with three levels (0 = baseline, 1 = after 1-month, 3 = after 3-month ICS/LABA treatment), treatment defined as a categorical variable with two levels. To analyze individual time-points, a paired Student’s t-test was used. All analyses were done in SPSS (version 22).

### Gene-expression analyses in the FAIR study

Gene-expression data analysis was conducted using the statistical package R (version 3.2.2). We investigated the association between nasal epithelial gene-expression and RV/TLC% predicted at screening of patients in the FAIR study (n = 76). To this end, a linear regression analysis was performed adjusting for smoking status as a possible confounder. Ge_ij_ represents the log2 gene expression value for a gene in sample i from patient j, ɛ_ij_ represents the error that is assumed to be normally distributed:

$$Ge_{ij} = \beta_{0} + \beta_{1} X_{Smoking\;Status - i} + \beta_{2} X_{RV/TLC\% \;predicted - i} + \varepsilon_{\text{ij}}$$

Genes increased with higher RV/TLC% predicted will be hence forth referred to as the hyperinflation gene-expression signature. The model used for the gene-expression analyses are explained in detail in the online supplementary materials. A false discovery rate (FDR) < 0.25 calculated using the Benjamini Hochberg (BH) correction was used for all analyses.

### Comparison of the hyperinflation gene-expression signature to the lower airways

We next compared the hyperinflation gene-expression signature in nasal epithelium to a RV/TLC% predicted signature generated in bronchial biopsies in the Groningen and Leiden Universities study of Corticosteroids in Obstructive Lung Disease (GLUCOLD) study^13^. The GLUCOLD study recruited male and female patients from family practices in Groningen and Leiden in the Netherlands. Exclusion criteria included asthma history or diagnosis and inhaled corticosteroid use within 6 months prior to randomization. Patients were randomly assigned to one of four treatment arms:

1. 6-month fluticasone propionate 500 μg b.i.d. followed by placebo for 24 months.
2. 30-month fluticasone propionate 500 μg b.i.d.
3. 30-month combination therapy with fluticasone/salmeterol 500/50 μg b.i.d.
4. 30-month placebo.

In all patients, a spirometry was performed every 3 months. In addition, bronchoscopy with endobronchial biopsies, sputum induction, and methacholine challenge were performed at 0, 6 and 30 months. During bronchoscopy, six macroscopically adequate bronchial biopsy specimens were immediately fixed in 4% neutral buffered formalin for 24 h, then processed and embedded in paraffin, and two biopsies were immediately snap frozen and stored at − 80 °C for RNA studies. Of the 114 patients enrolled, 101 were treatment-compliant. Of these, 88 and 77 have snap frozen biopsies available for two-time points (baseline and 6 months) or three-time points (baseline, 6 months and 30 months).

A linear model was used to compare association between gene expression and RV/TLC% predicted correcting for current smoking status. The t-statistic generated from this analysis was used to rank the genes associated with RV/TLC in the lower airways. Gene set enrichment analysis (GSEA) (v 2.2.2) was used to compare this ranked list to the hyperinflation gene-expression signature generated in the nose. For all GSEA analyses, a false-discovery rate threshold of FDR less than 0.05 was used to determine significant enrichment.

### Comparison of the hyperinflation gene-expression signature between COPD patients and non-COPD controls

To compare nasal epithelium from COPD patients to non-COPD controls, a subset of the COPD patients included in the FAIR study collected at the UMCG (at screening), were compared to nasal epithelium of age matched healthy patients collected at the same location during the same time as part of the Study to Obtain Normal Values of Inflammatory Variables From Healthy Subjects (NORM) study (NCT00848406). In the NORM study, healthy smokers and never-smokers were included, defined by an absence of respiratory symptoms (no bronchial hyperresponsiveness to methacholine and a normal spirometry with FEV_1_/FVC > 70% and FEV_1_ > 80% predicted). All nasal brushes were extracted and run on Affymetrix-Hugene-st1.0 arrays at the same time to minimize technical variation between the studies. A linear model was used to compare COPD and healthy controls correcting for age, gender and current smoking status. The t-statistic generated from this analysis was used to rank the genes associated with COPD. The hyperinflation genes were compared with these ranked genes. This analysis was repeated in two additional COPD related signatures from both the large and small airways using two publically available independent datasets (GSE37147 and GSE56341)^14,15^.

### Large airway epithelium

To examine whether the hyperinflation gene-expression signature was associated with the presence of COPD in large airway bronchial brushes, we utilized a dataset which we have previously published of large airway bronchial brushes from COPD and non-COPD patients (GSE37147)^14^. Using spirometry measurements obtained within 1 year of bronchoscopy, COPD was defined as the presence of both an FEV1/FVC ≤ 0.7 and FEV1% predicted < 80. The data was analysed comparing COPD to non-COPD using a linear model adjusting for age, gender, smoking status (current or former smoker), and cumulative tobacco exposure (calculated for the time of bronchoscopy). Genes were ranked according to the generated t-statistic from this analysis, then GSEA was used to compare this ranked list to the hyperinflation gene-expression signature.

### Small airway epithelium

To examined whether the hyperinflation gene-expression signature was associated with the presence of COPD in small airway bronchial brushes, we utilized a publically available dataset of small airway bronchial brushes from COPD patients (GOLD stage II and III) and healthy controls (GSE56341)^15^. Raw data was obtained from the Gene Expression Omnibus (GEO) and was normalized using RMA. Data was analyzed using limma comparing COPD patients (GOLD stage II and III) to healthy controls in small airway brushings. Genes were ranked according to the generated t-statistic from this analysis, then GSEA was used to compare this ranked list to hyperinflation gene-expression signature.

### Comparison of the hyperinflation gene-expression signature with ICS/LABA treatment

Next, we investigated whether the hyperinflation gene-expression signature was altered during ICS/LABA treatment. To this end, we compared gene-expression changes after increasing the relative dose of ICS/LABA treatment (BDP/F and BUD/F combined) in the FAIR study (baseline compared the 3-months), using a linear mixed effect model. The model was adjusted for smoking status, age and gender as possible confounders. The t-statistic generated from this analysis was used to rank the genes associated with changes in expression during 3 months high dose ICS/LABA. GSEA was used to compare this ranked list to the hyperinflation gene-expression. Finally, to verify the above analysis in the lower airways we investigated how the hyperinflation gene-expression signature responded to 6-months ICS or ICS/LABA compared to placebo in bronchial biopsies (GLUCOLD). A linear mixed effect model was conducted comparing the interaction between the change in expression over 6 month treatment of ICS or ICS/LABA to placebo, adjusting for smoking status, age and gender as possible confounders. The t-statistic generated from this analysis was used to rank the genes associated with changes in expression during 6 months ICS/LABA or ICS compared to placebo. GSEA was used to compare this ranked list to the hyperinflation gene-expression.

### Pathway analysis

Pathway analysis was conducted using Gene Network analysis. This pathway analysis software uses an independent gene expression dataset of ~ 78,000 samples to predict the function of genes in an unbiased way. We used this method to predict (currently unknown) gene functions based on known biological pathways available in the molecular signatures database MSigDB (https://www.broadinstitute.org)^16^.

## Results

### Patient demographics

One hundred and seventeen patients entered the screening period from whom nasal epithelial brushes were available in 76 cases. Because the strict inclusion criteria accounted for a much higher than expected screening failure rate and a much lower than planned recruitment pace, the FAIR study was terminated prematurely resulting in lower numbers than expected for each treatment group. A total of 60 COPD patients were randomized, with 29 patients assigned to BDP/F and 31 patients to BUD/F. After randomization, 20 patients dropped out due to an exacerbation and 40 completed the study. A total of 57 out of the 60 randomized COPD patients had upper airway epithelial brushes available at one or more time points with RNA of sufficient quality.

### The effect of increasing the dose of ICS/LABA treatment on lung function parameters over the course of 3 months

**Figure 2:**
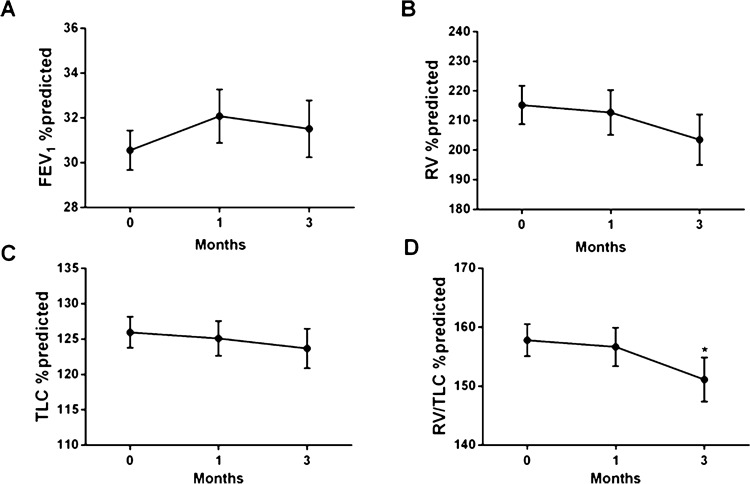
Influence of ICS/LABA (BDP/F and BUD/F treatment arms combined) on measures of hyperinflation and lung function. Lung function measurements were taken before study medication at baseline, 1 month and 3 months. (**A**) FEV_1_% predicted, (**B**) RV% predicted (**C**) TLC% predicted (**D**) RV/TLC% predicted and **E**) FEF 25–75% predicted. Linear mixed effects model comparing each time point. *p < 0.05 FEV_1_, forced expiratory volume in one second; RV, residual volume; TLC, total lung capacity; RV/TLC, residual volume/total lung capacity; FEF 25–75% predicted, the average forced expiratory flow during the mid (25–75%) portion of the FVC. All data are expressed as mean ± SEM.

We found no significant effect of increasing the dose of ICS/LABA treatment on pre- and post-dose RV% predicted (the primary end point measured in this clinical trial), TLC% predicted, and FEV_1_% predicted over the 3-month treatment period (Fig. 2A–C). However, pre-dose RV/TLC% predicted significantly improved compared to baseline following 3-month treatment (Fig. 2D, p < 0.05). Interestingly, when analyzing the individual time points (paired Student’s t-test) we did not find any changes after 1 month but did so after 3 months, suggesting that a longer period of treatment is required for the improvement in RV/TLC% predicted to occur. This improvement in RV/TLC% predicted after 3-months of treatment was not accompanied by a significant improvement of clinical symptoms measured by CCQ scores and Transition Dypsnae Index (TDI) (Table 1).

**Table 1:** Patient characteristics (BDP/F and BUD/F combined).

	Screening	Baseline	Month 1	Month 3
Number of patients	117	60	50	40
Male/female, n	83/34	44/16	39/11	31/9
Age, years	63.1 (8.7)	62.1 (8.5)	62.9 (8.6)	62.8 (8.9)
BMI kg/m^2^	25.5 (4.7)	24.8 (3.8)	24.9 (3.5)	24.8 (3.7)
Current smokers, n (%)	39 (33)	21 (35)	17 (34)	14 (35)
FEV_1_% predicted pre	34.3 (7.8)	30.6 (6.0)	32.1 (7.3)	31.5 (6.8)
Post 2 h		37.9 (8.3)	37.4 (8.2)	36.7 (7.6)
RV% predicted pre	211.9 (58.8)	215.2 (49.6)	212.7 (53.0)	203.5 (51.9)
Post 2 h		187.4 (47.7)	191.8 (50.9)	188.1 (51.2)
TLC% predicted pre	127.3 (20.5)	126.0 (16.9)	125.1 (17.3)	123.7 (16.9)
Post 2 h		122.6 (17.0)	123.9 (17.9)	123.3 (17.6)
RV/TLC% predicted pre	152.4 (23.6)	157.8 (21.0)	156.7 (22.7)	151.1 (22.9)*
Post 2 h		140.9 (22.4)	142.4 (22.5)	140.3 (22.9)
CCQ		1.95 (0.79)		2.04 (0.87)
BDI/TDI		6.04 (1.61)		0.08 (2.53)

Lung function measurements were taken before and 2 h after study medication at screening, baseline, 1 month and 3 months. Student paired T-test compared to baseline * < 0.05. FEV_1_, forced expiratory volume in one second; RV, residual volume; TLC, total lung capacity; RV/TLC, residual volume/ total lung capacity; CCQ, Clinical COPD Questionnaire; BDI, Baseline Dyspnea Index, TDI, Transition Dyspnea Index. All data are expressed as mean ± SD.

### Associations between upper airway gene-expression and hyperinflation

**Figure 3:**
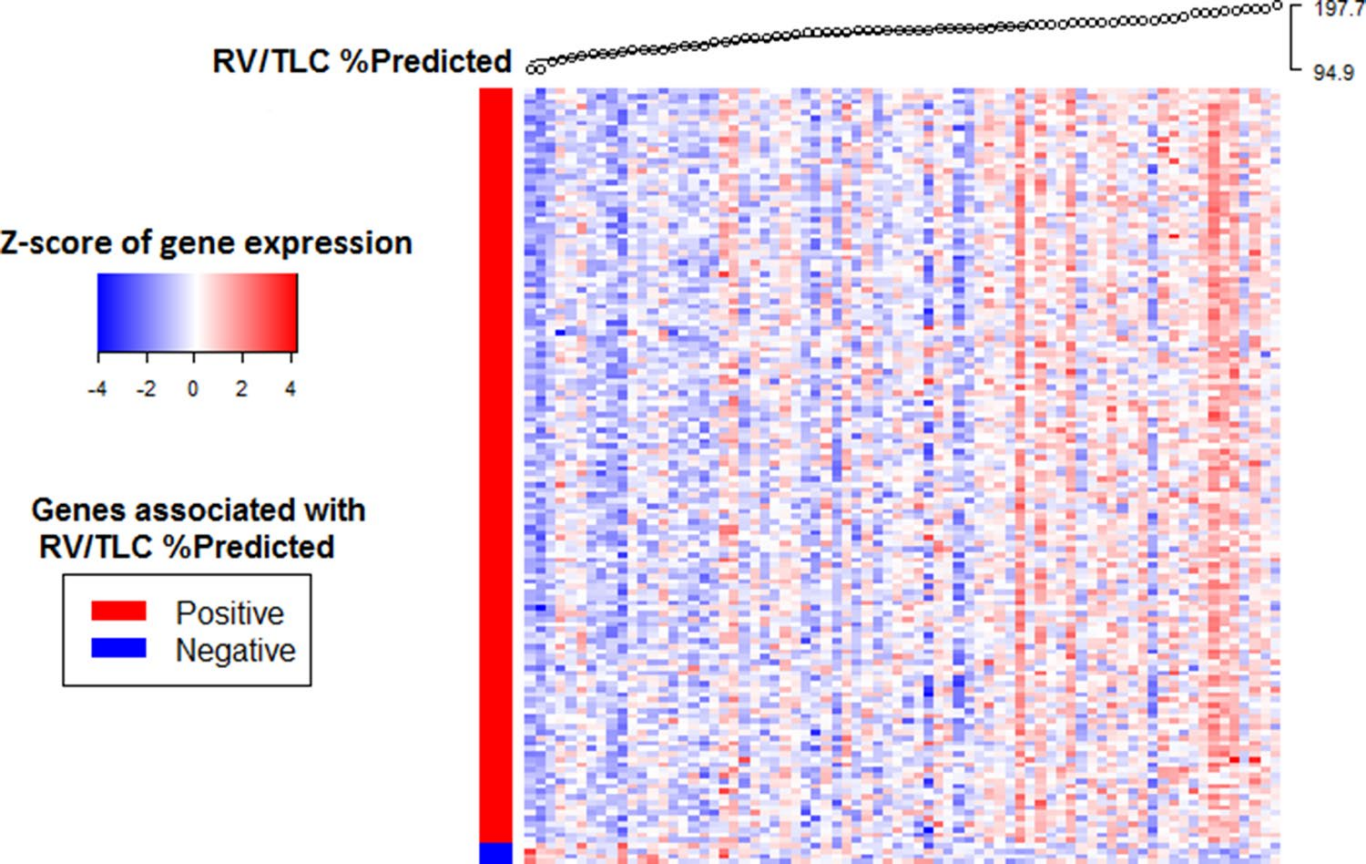
Nasal gene-expression associated with RV/TLC% predicted. Heatmap of genes associated with RV/TLC% predicted at baseline in all screened patients with severe COPD (FDR < 0.25).

We identified 140 genes for which the expression in nasal epithelium was increased with more severe hyperinflation as measured by RV/TLC% predicted and 4 genes for which expression was decreased (n = 76, FDR < 0.25). A list of the top 50 genes is presented in Table 2. Figure 3 shows a heatmap of the 144 genes associated with RV/TLC% predicted with patients ordered based on the severity of hyperinflation.

**Table 2:** List of top 50 genes associated with RV/TLC% predicted (FDR < 0.25).

Gene	T value	P value	FDR
RLN1	− 3.759	0.000347	0.189
ABHD16A	3.645	0.000506	0.195
TMEM104	3.648	0.000501	0.195
SLC22A18	3.649	0.000499	0.195
SLC6A6	3.657	0.000487	0.195
SIK3	3.669	0.000468	0.195
USP5	3.669	0.000468	0.195
TAB1	3.671	0.000464	0.195
LDOC1L	3.691	0.000434	0.195
ZNF142	3.694	0.00043	0.195
ABCB6	3.696	0.000427	0.195
PAK6	3.699	0.000424	0.195
SRRM2	3.719	0.000396	0.195
KHSRP	3.745	0.000364	0.189
HCFC1	3.752	0.000355	0.189
SMARCA4	3.777	0.000327	0.184
VAC14	3.785	0.000319	0.184
CTSD	3.798	0.000304	0.182
ATP13A1	3.828	0.000275	0.170
CBX6	3.847	0.000258	0.164
BAG6	3.850	0.000256	0.164
RPTOR	3.860	0.000248	0.164
PPRC1	3.861	0.000246	0.164
CARM1	3.863	0.000245	0.164
ALDOA	3.875	0.000235	0.164
SEPT9	3.891	0.000223	0.164
ARF3	3.922	0.000201	0.164
ATF6B	3.925	0.000198	0.164
WDR18	3.943	0.000186	0.164
ATN1	3.963	0.000174	0.164
DAG1	3.980	0.000164	0.162
UBE2O	3.985	0.000161	0.162
VARS	3.995	0.000156	0.162
TNPO2	4.043	0.000132	0.154
MECP2	4.065	0.000123	0.151
PRRC2A	4.093	0.000111	0.146
DNMBP	4.120	0.000101	0.142
PCNXL3	4.131	9.72E−05	0.142
MYH14	4.134	9.64E−05	0.142
EXOC7	4.134	9.63E−05	0.142
MINK1	4.169	8.53E−05	0.142
MEPCE	4.172	8.43E−05	0.142
MAVS	4.236	6.72E−05	0.142
SAMD4B	4.277	5.80E−05	0.142
UBA1	4.380	4.02E−05	0.132
RNF123	4.441	3.22E−05	0.127
GEMIN4	4.497	2.62E−05	0.127
ANKRD52	4.608	1.75E−05	0.115
G6PD	4.726	1.13E−05	0.111
SRCAP	4.806	8.34E−06	0.111

### The hyperinflation signature in the upper airways is representative of the gene-expression signature related to hyperinflation in the lower airways

**Figure 4:**
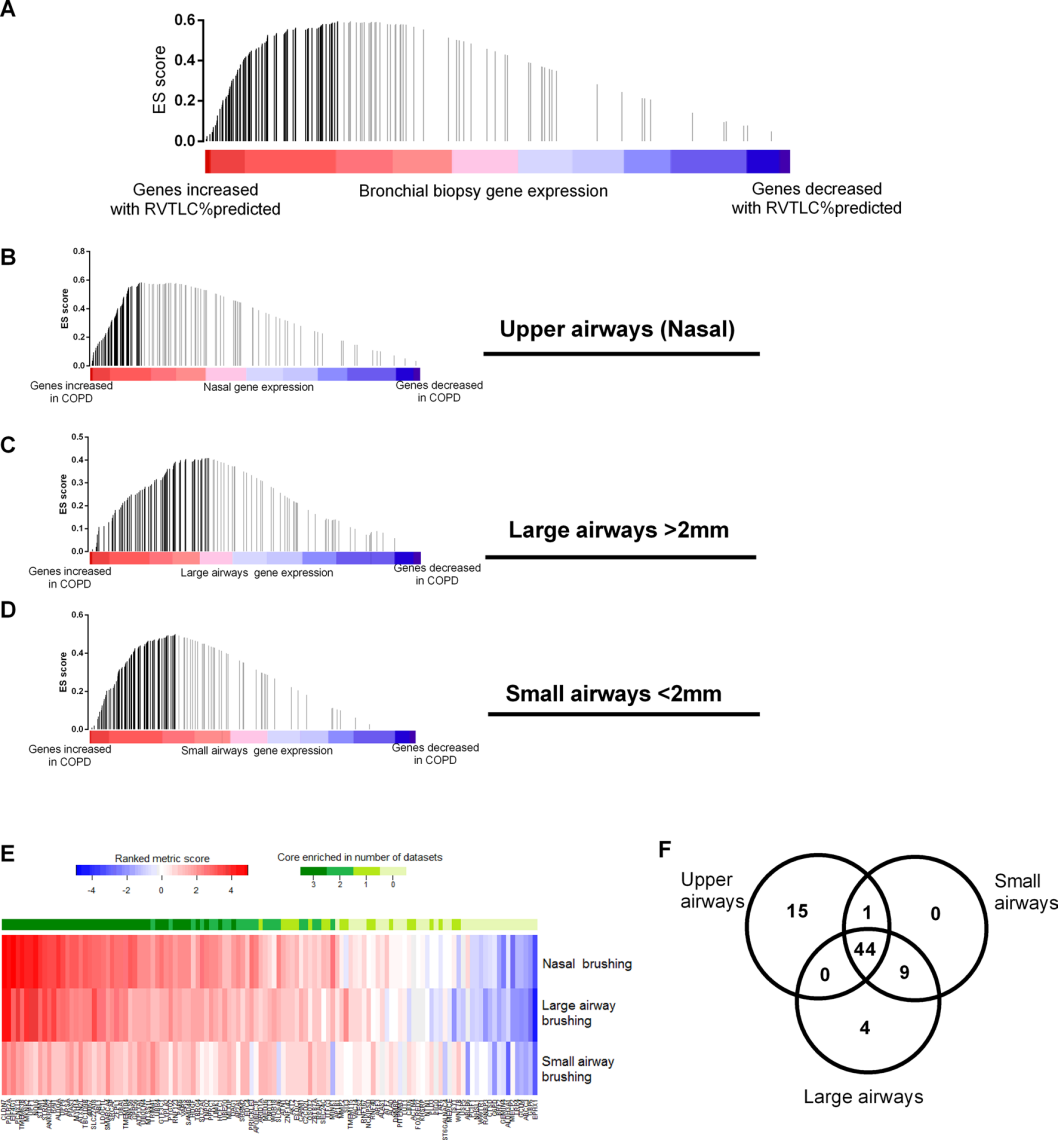
The relationship between the hyperinflation gene expression signature and presence of COPD in the upper, large and small airway brushings. (**A**) Enrichment of genes increased with RV/TLC% predicted in the upper airway among genes increased with RV/TLC% in bronchial biopsies (p < 0.001). The color bar indicates the genes ranked according to their association with RV/TLC% predicted in bronchial biopsies (blue representing a negative association with RV/TLC% predicted and red indicating a positive association). Enrichment of genes increased with RV/TLC% in the nose among genes increased in COPD (**B**) nasal epithelium, (**C**) large airway brushings and (**D**) small airway brushings, compared to healthy controls (GSEAp < 0.001). The color bar indicates the genes ranked according to their expression in COPD epithelial cells compared to healthy/non-COPD controls (blue representing genes decreased in COPD epithelium compared to healthy/non-COPD controls, while red indicates an increase in epithelial gene-expression). (**E**) Heatmap of rank metric score of the hyperinflation signature GSEA in comparison to the upper, large and small airway brushing from COPD and non-COPD controls. The enrichment of genes in the hyperinflation signature among genes increased in COPD in the upper, large and small airway brushing is represented with the green scale and illustrated in a (**F**) Venn diagram.

In order to evaluate whether the hyperinflation gene-expression signature in the nasal epithelium is representative for the lower airways, we performed a GSEA on gene-expression data derived from bronchial biopsies of COPD patients who participated in the GLUCOLD study^13^. Their clinical characteristics are summarized in Table 3. Figure 4A shows a strong overlap between genes increased with RV/TLC% predicted in nasal epithelium and genes increased with RV/TLC% predicted in bronchial biopsies (GSEA p < 0.05).

**Table 3:** GLUCOLD patient characteristics.

	6-months Fluticasone	30-months Fluticasone	30 months Fluticasone/salmeterol	Placebo
N	21	19	18	21
Male/female	18/3	16/3	15/3	17/4
Age (years), mean (SD)	63.7 (7.6)	60.8 (7.9)	60.8 (8.6)	58.4 (8.1)
Current smokers, N	10	10	12	14
FEV_1_% pred, mean (SD)	64.7 (8.6)	65.2 (8.5)	61.9 (9.6)	61.3 (8.8)
RVTLC % pred	124.7 (19.1)	120.1 (18.6)	126.4 (19.3)	125.2 (16.9)

FEV_1_, forced expiratory volume in one second; RV/TLC, residual volume/ total lung capacity. All data are expressed as mean ± SD.

### Relationship between the hyperinflation gene-expression signature and presence of COPD throughout the respiratory tract

We next examined whether the hyperinflation gene-expression signature was associated with the presence of COPD in both the nasal and lower airway epithelium. To run this analysis in the (i) nasal epithelium, a subset of COPD nasal gene expression profiles from the FAIR study were compared to age matched healthy subjects from the NORM study; while the comparisons between COPD and non-COPD controls in the (ii) large and (iii) small airways was conducted in two publically available datasets (GSE37147 and GSE56341)^14,15^. By GSEA, we found similar enrichment of the hyperinflation gene-expression signature among genes increased with COPD compared to non-COPD in upper, large and small airway epithelial brushings (all GSEA p < 0.05, Fig. 4B–D).

Interestingly, we found a large overlap of the core enriched genes associated with our hyperinflation gene-expression signature in all three compartments of the airways indicating a common signature throughout the respiratory tract (Fig. 4E,F). Table 4 shows an overview of the principal characteristics from the main datasets used.

**Table 4:** Principal characteristics from the main datasets used (FAIR, GLUCOLD, NORM).

	FAIR	GLUCOLD	NORM
Number of patients, n	60	79	77
Male/female, n	44/16	66/13	41/36
Age, years	62.1 (8.5)	60.9 (8.1)	36.1 (16.2)
Current smoker, n (%)	21 (35%)	46 (58%)	41 (53.2%)
FEV_1_% predicted	37.9 (8.3)	63.3 (8.9)	108.1 (10.5)
RV % predicted	187.4 (47.7)	N/A	93.7 (17.5)
TLC % predicted	122.6 (17.0)	N/A	104.0 (9.4)
RV/TLC % predicted	140.9 (22.4)	124.1 (18.5)	85.6 (12.4)

All data are expressed as mean ± SD.

FEV_1_, forced expiratory volume in one second; RV, residual volume; TLC, total lung capacity; RV/TLC, residual volume/ total lung capacity.

### The effect of ICS/LABA treatment on the hyperinflation gene-expression signature

To evaluate whether the ICS/LABA treatment alters genes related to hyperinflation, we performed a GSEA on the change of gene-expression associated with doubling the dose of ICS/LABA (BDP/F and BUD/F groups combined), between baseline and 3 months treatment. Figure 4A shows that genes increased with hyperinflation were significantly enriched among genes that were decreased by doubling the dose of ICS/LABA (GSEA p < 0.05).

**Figure 5:**
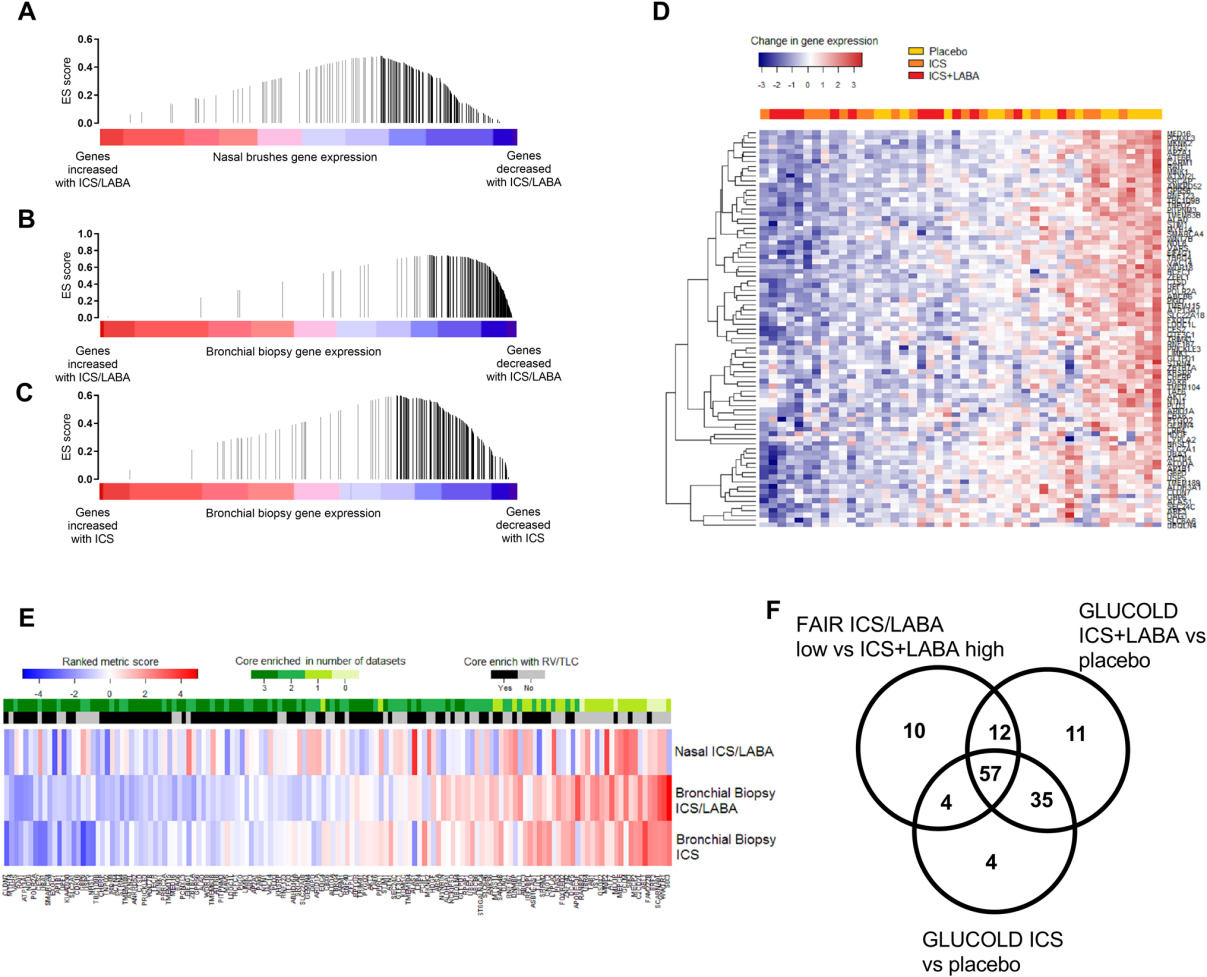
Gene set enrichment analysis (GSEA) comparison of genes associated with RV/TLC% predicted in bronchial biopsies (GLUCOLD). (**A**) Enrichment of genes increased with RV/TLC% predicted in the nose among genes down-regulated in nasal epithelium following increased dosage with ICS/LABA (p < 0.001) or treatment with (**B**) ICS/LABA (fluticasone/salmeterol) (p < 0.001) and (**C**) solely ICS (fluticasone) (p < 0.001) for 6 months compared to placebo in bronchial biopsies. The color bar indicates the genes ranked according to their change in expression with ICS (blue representing a treatment-induced decrease and red an increase in gene-expression). The vertical bars in all plots indicate the genes increased with RV/TLC% predicted with the location of the bar indicating the occurrence of that gene within the ranked gene list and the height of the bars indicating the running GSEA enrichment score (black bars = core enriched genes, grey bars = core non-enriched genes). (**D**) Heatmap of the change of expression of genes associated with RV/TLC% predicted core enriched to be decreased in bronchial biopsies from patients with COPD after 6 months treatment with ICS/LABA (fluticasone/salmeterol) or solely ICS (fluticasone). (**E**) Heatmap of rank metric score of the RV/TLC signature GSEA comparison to increased dosage with ICS/LABA (p < 0.001) in nasal epithelium, and treatment with ICS/LABA (fluticasone/salmeterol) or solely ICS (fluticasone) compared to placebo in bronchial biopsies. The enrichment of genes in the RVTLC signature among genes decreased within the three datasets is represented in the green scale and illustrated in a (**F**) Venn diagram.

To validate the above hypothesis in the lower airways the hyperinflation gene-expression signature was subsequently investigated in the GLUCOLD study where genome-wide gene-expression data was available in bronchial biopsies from COPD patients before and after 6-month treatment with ICS (Fluticasone) ± LABA (Salmeterol) compared to placebo. The ICS/LABA and ICS arms were compared to placebo independently to distinguish the overall effect of ICS from the combined treatment. Figure 5B demonstrates that genes increased with hyperinflation in nasal epithelium (FAIR study) were significantly enriched among genes that were decreased by ICS/LABA compared to placebo in the bronchial biopsies (GLUCOLD study, GSEA p < 0.05), mirroring the results seen in nasal epithelium. Finally, 6-month treatment with ICS compared to placebo revealed similar results as ICS/LABA (GSEA p < 0.05), suggesting ICS treatment affected the hyperinflation gene-expression profile in the absence of LABA (Fig. 5C). Figure 5D shows a heatmap of the genes in the hyperinflation gene-expression profile which were core enriched among genes decreased in both the ICS and ICS/LABA treatment groups compared to placebo. We found a large overlap of the core enriched genes associated with our hyperinflation gene-expression signature in all three treatment groups in the airways indicating a common subset of hyperinflation related genes which are repressed by ICS ± LABA treatment in both the upper and lower airways (Fig. 5E,F).

### Pathway analysis

Pathway analysis was conducted on the hyperinflation gene-expression profile during screening. This analysis identified that “DNA damage response, signal transduction by p53 class mediator resulting in induction of apoptosis” and “histone deacetylation” pathways were increased with increasing severity of hyperinflation. The list of the top 10 pathways is displayed in Table 5.

**Table 5:** Pathway analysis.

Pathway or process	p-value
Signal transduction by p53 class mediator resulting in induction of apoptosis	1 × 10^–27^
DNA damage response, signal transduction by p53 class mediator resulting in induction of apoptosis	1 × 10^–26^
Histone deacetylation	4 × 10^–26^
Protein targeting	3 × 10^–22^
Regulation of histone deacetylation	5 × 10^–22^
Positive regulation of protein deacetylation	1 × 10^–21^
Protein deacetylation	7 × 10^–21^
In utero embryonic development	3 × 10^–20^
Regulation of protein deacetylation	5 × 10^–20^
rRNA transcription	7 × 10^–20^

List of the top 10 pathways associated with the hyperinflation gene signature.

## Discussion

In this randomized double-blind study, increasing the equivalent dose of ICS/LABA did not improve FEV_1._ However, it did improve hyperinflation as reflected by RV/TLC% predicted, while it took up to 3 months for the effect to occur. Furthermore, we identified a nasal epithelial gene-expression signature that associated with hyperinflation as reflected by RV/TLC% predicted and validated this signature in bronchial biopsies of an independent cohort of patients with mild-to-moderate COPD. Finally, we found this signature to be increased in COPD, both in epithelium derived from the upper airways, and of the lower large and small airways, indicating a common signature associated with hyperinflation throughout the respiratory tract. Our study suggests that this nasal epithelial gene-expression profile can serve as a non-invasive biomarker of the severity of hyperinflation in COPD as reflected by RV/TLC% predicted.

We found this hyperinflation associated nasal gene expression signature to be dynamic with ICS/LABA treatment in COPD. Furthermore, this change was concordant in the lower airways after ICS/LABA treatment in an independent study using bronchial biopsies. Genes involved in p53-mediated apoptosis were particularly enriched in this hyperinflation signature.

When analyzing the nasal gene-expression signature in an independent dataset of bronchial biopsies derived from the lower large airways of patients with COPD, we found a similar association with the severity of hyperinflation, even though the degree of hyperinflation in this cohort of patients wild mild-to-moderate COPD was on average less. This observation is compatible with the field-of-injury hypothesis put forward by Gower et al*.,* proposing that inhalation of environmental ‘inhaled exposures can elicit a common molecular response throughout the respiratory tract’^12^.

We did not find an improvement in FEV_1_% predicted, with 3-month ICS/LABA treatment. However, we did find a statistically significant improvement in RV/TLC% predicted with 3-month treatment. This improvement was not present after 1-month of treatment, suggesting that a treatment for a longer period is required before an effect can be seen. Similar to our findings, O’Donnell et al*.* showed an improvement in hyperinflation, as reflected by RV and Forced Respiratory Capacity (FRC), following treatment with fluticasone/salmeterol for 2 months, while John et al. showed a significant decrease in RV/TLC% predicted after 3-month treatment with extra-fine particle beclomethasone^9,10^. Further, larger-sized studies have to assess whether either short-term treatment with small or large particle ICS/LABA combination, or ICS alone, differentially affect hyperinflation. Also, it should be evaluated whether the effect of ICS treatment continues to improve with longer duration.

We established that the RV/TLC% predicted gene-expression signature is different in COPD patients compared to healthy controls. This was the case not only in the upper, but also the large and small airway epithelium, providing suggestive evidence that the identified genes are not only relevant to hyperinflation per se, but also to COPD pathogenesis. However, given the cross-sectional nature of our study we do not elucidate whether this is a cause or consequence of hyperinflation or a coincidental finding by a common pathogenetic mechanism leading to increased severity of hyperinflation. Prospective studies in less severe COPD patients have to reveal this.

Interestingly, we show that the gene-expression signature for hyperinflation was enriched among genes that were affected after doubling the dose of ICS/LABA. Analysis of an independent study of bronchial biopsies showed that 6-month treatment with ICS on the hyperinflation signature provided comparable results in gene-expression as ICS/LABA combination^13^, which may suggest that the response was mainly driven by the ICS component rather than the LABA component. As ICS treatment was found to inhibit the expression of p53 pathway genes collectively in the current study, our results suggest that ICS may inhibit smoke-induced apoptosis by repressing the p53 pathway. Alternatively, ICS may have an effect on (epithelial) genes that affect inflammation indirectly^13^. Reduction of peripheral inflammation can contribute to the reduction of small airways narrowing and reduction in peribronchiolar inflammation in improvement of peribronchial contractile forces, all leading to improvement of hyperinflation. Our findings suggest that the nasal gene expression signature identified in this study may be used as a biomarker for changes in inflammation which result in improvement in RV/TLC% predicted.

One of the main strengths of this study is the use of independent cohorts (1) to validate the gene-expression signature associated with RV/TLC% predicted as found in the nose in the signature present in the large airways (GLUCOLD) and (2) to confirm that this signature was differentially expressed in COPD both in upper airway epithelium and throughout the respiratory tract. Furthermore, our study consists of a unique cohort of patients with both severe COPD and a significant degree and broad range of hyperinflation. There are some limitations to this study. The subgroups of current and former smokers were too small to do independent analysis on the influence of smoking status on both the response to ICS/LABA therapy and nasal gene expression. Furthermore, we were unable to directly compare the nasal and small airways gene-expression profile in the same patients due to technical difficulties obtaining samples from the lower airways in severe COPD. However, we were able to show that our hyperinflation signature was increased in independent large and small airway epithelial bushings from mild-to-moderate COPD patients, providing evidence that this signature is altered throughout the respiratory tract. Another limitation is the lenient FDR < 0.25 that we used to identify our hyperinflation gene-signature in the nasal epithelium. Despite this fact, our signature was associated with RV/TLC% predicted in independent dataset of large airway bronchial biopsies from mild-to-moderate COPD patients, demonstrating the robustness of this signature. Finally, as a placebo arm was not included in this study, we were unable to account for a placebo effect on the clinical measurements in this study.

## Conclusion

In summary, treatment with ICS/LABA in patients with severe COPD decreased hyperinflation as measured by RV/TLC% predicted after 3-month treatment. This improvement in hyperinflation was not accompanied by an improvement in respiratory symptoms. We did not find any change after 1 month of ICS/LABA treatment, suggesting that a longer treatment period is required for the improvement to occur. Further, we have identified and validated a gene-expression signature that is associated with RV/TLC% predicted in upper airway epithelium from patients with severe COPD, a clinically relevant phenotype that drives dyspnea in COPD. We found this signature to associate with increased p53 mediated apoptosis. Since ICS/LABA treatment improves RV/TLC% predicted and shifts the associated gene-expression signature towards normal, our findings provide a clear gene signature associated with ICS-induced clinical improvement in RV/TLC% predicted in COPD.

## Supplementary information

Supplementary Information 1.

## Data Availability

The datasets used and/or analyzed during the current study are available from the corresponding author on reasonable request.
