# Supplementary material for: Identifying a nasal gene expression signature associated with hyperinflation and treatment response in severe COPD
Source: Sci Rep. 2020 Oct 15;10:17415. doi: 10.1038/s41598-020-72551-0 (PMC7562702; doi:10.1038/s41598-020-72551-0)
Supplement: Supplementary file 1 — Supplementary Information 1. [file 41598_2020_72551_MOESM1_ESM.docx]

**Title:** Identifying a nasal gene expression signature associated with hyperinflation and treatment response in severe COPD

**Authors:** Alen Faiz^1,2,3‡^, Kai Imkamp^1,2‡*^, Erica van der Wiel^1,2^, Ilse M Boudewijn^1,2^, Gerard H Koppelman^2,4^, Corry-Anke Brandsma^2,5^, Huib AM Kerstjens^1,2^, Wim Timens^2,5^, Sebastiaan Vroegop^6^, Henk R Pasma^7^, Wim G Boersma^8^, Pascal Wielders^9^, Frank van den Elshout^10^, Khaled Mansour^11^, Katrina Steiling^12^, Avrum Spira^12^, Marc E Lenburg^12^, Irene H Heijink^2,3^, Dirkje S Postma^1,2^ and Maarten van den Berge^1,2^

**Affiliations:**

^1^University of Groningen, University Medical Center Groningen, Department of Pulmonology, Groningen, The Netherlands

^2^University of Groningen, University Medical Center Groningen, GRIAC (Groningen Research Institute for Asthma and COPD), Groningen, The Netherlands

^3^University of Groningen, University Medical Center Groningen, Department of Pathology & Medical Biology*,* section Medical Biology, Groningen, The Netherlands

^4^ University of Groningen, University Medical Center Groningen, Department of Pediatric Pulmonology and Pediatric Allergology, Beatrix Children's Hospital, Groningen, The Netherlands.

^5^University of Groningen, University Medical Center Groningen, Department of Pathology and Medical Biology, Groningen, the Netherlands

^6^Martini Hospital Groningen, Department of Pulmonary Diseases, Groningen, the Netherlands

^7^Medical Center Leeuwarden, Department of Pulmonary Diseases, Leeuwarden, the Netherlands

^8^Medical Center Alkmaar, Department of Pulmonary Diseases, Alkmaar, the Netherlands

^9^Catharina Hospital Eindhoven, Department of Pulmonary Diseases, Eindhoven, the Netherlands

^10^Rijnstate Hospital, Department of Pulmonary Diseases, Arnhem, the Netherlands

^11^Orbis Concern, Department of Pulmonary Diseases, Sittard, the Netherlands

^12^ Boston University School of Medicine, Division of Computational Biomedicine, Department of medicine, Boston, Massachusetts, USA

**Supplementary materials**

*Patient population*

The FAIR study included patients when they fulfilled the following inclusion criteria at the screening visit: age ≥ 40 years, a post-bronchodilator FEV1/FVC < 0.7 and FEV1 < 50% of the predicted normal value, a smoking history of at least 10 pack-years, regular use of bronchodilators, a Functional Residual Capacity (FRC) > 120% of predicted, and a Baseline Dyspnea Index (BDI) total score ≤ 10. We excluded patients with a diagnosis of asthma or other clinically or functionally relevant respiratory disorders (other than COPD), pregnant or lactating women, patients with clinically unstable concurrent disease, patients with narrow-angle glaucoma, clinically significant laboratory and ECG abnormalities, patients with COPD exacerbations and/or symptomatic infection of the airways requiring antibiotic therapy (at least 5 days) in the 2 months prior to screening and during the study period, patients requiring long term (> 12 hours daily) oxygen therapy for chronic hypoxemia, patients treated with depot corticosteroids in the 2 months preceding visit 1 and during the run-in period, patients with known allergy, sensitivity or intolerance to sympathomimetic drugs or inhaled corticosteroids or to any of the excipients contained in the study drugs, patients who had evidence of alcohol or drug abuse, not compliant with the study protocol or not compliant with the study treatments according to investigator’s judgment, major surgery in the previous 3 months and during the trial which could affect patient’s compliance in the study procedures, participation in another clinical trial with an investigational drug in the 2 months preceding visit 1 and patients requiring chronic mechanical ventilation for COPD.

*Sample size calculation*

Sample size was based on feasibility. As a total of 170 patients was planned to be available for recruitment and anticipating a screen failure rate of 15%, 144 patients were to be randomized. Estimating a non-evaluable rate of 10%, a total of 128 patients (64 per treatment group) could have been part of the ITT population. This anticipated number of 64 ITT evaluable patients per group had 80% power to detect a difference between treatments in the primary efficacy variable of 0.5 L (absolute value), by assuming a standard deviation (σ) of 1.0 L and by using a two-sample t-test with two-sided significance level of 0.05.

*Randomisation*

The computer generated randomisation list and the treatment code envelopes will be prepared by CROS NT S.r.l.; the codes will report a number ranging from 001 to 240.

Starting from the lowest number provided, in each site, at the end of the run-in period, patients will be sequentially assigned to the next study number, following the order in which they present themselves for the study.

Individual patient treatment code envelopes will be provided by CROS NT S.r.l. to the investigators; CROS NT S.r.l. will keep a master code list.

The investigator will keep the treatment code envelopes in a locked, secure storage facility.

A treatment code envelope can only be opened in an emergency situation where the investigator considers it essential to know which treatment the subject was taking. If possible, it is recommended that the investigator contacts the Sponsor before opening an envelope. The monitor shall be promptly notified when a treatment code envelope is opened. The investigator shall provide a certified explanation of why the treatment code was opened in the electronic Case Report Form for the subject or directly on the opened treatment code envelope.

All treatment code envelopes, either unopened or opened, will be returned to Chiesi Farmaceutici S.p.A. upon termination of the study.

*RNA Isolation and Size Fractionation*

Nasal brushes were immediately snap-frozen and stored at -80 ^o^C. RNA was extracted from nasal brushes and fractioned into low molecular weight (< 200 nt) and high molecular weight (> 200 nt) fractions, by using the miRNeasy mini kit (QIAGEN) according to manufacturer’s protocol. The purity of RNA fractions was checked on NanoDrop 1000 UV-Vis spectrophotometer and the integrity of large RNA fraction was assessed by running RNA Pico assay in the Agilent 2100 BioAnalyzer.

*Microarray hybridization*

All procedures were performed at Boston University Microarray Resource Facility as described previously in GeneChip® Whole Transcript (WT) Sense Target Labeling Assay Manual (Affymetrix, Santa Clara, CA, current version available at 24 www.affymetrix.com)^1^.

*Data acquisition, probeset summarization and normalization, and data preprocessing*

Normalization was performed with R statistical software V3.0.2. using Robust Multichip Analysis (RMA) sketch algorithm workflow. Microarray data quality was assessed using relative log expression (RLE) plots, normalized unscaled standard error (NUSE) plots. Based on the sample-wise global dissimilarity of gene expression data according to the RLE and NUSE plots, a total of 3 microarrays were excluded.

*Enrichment analysis*

Enrichment analysis of other gene expression datasets was performed using GSEA v2.2.2. Enrichment p-values were calculated by gene set permutation (n=1000), and significant enrichment was determined by an FDR-corrected p-value of < 0.05. The core enrichment genes, were defined by GSEA as the genes with the most contribution to the significant enrichment.

We examined whether the gene signature that was linked to RV/TLC% predicted was associated with the presence of COPD in both the upper airway epithelium and throughout the respiratory tract compared to non-COPD individuals.

**References**

1. van den Berge, M. *et al.* Airway gene expression in COPD is dynamic with inhaled corticosteroid treatment and reflects biological pathways associated with disease activity. *Thorax* **69**, 14–23 (2014).
